# Prevalence and Trends in Active Smoking Among Adults Living with HCV in the U.S. over the Last Decade: A Population-Level Analysis

**DOI:** 10.3390/jcm14186671

**Published:** 2025-09-22

**Authors:** Mohammad Alabbas, Jingyi Shi, Yuqi Guo, Hongke Wu, Ibukunoluwa Oshobu, Maria Castano, Walaa Mahmoud, Shreya Sengupta, Omar T. Sims

**Affiliations:** 1 Department of Gastroenterology, Hepatology and Nutrition, Cleveland Clinic, Cleveland, OH 44195, USA; amywu06@gmail.com (H.W.); castanm3@ccf.org (M.C.); mahmouw@ccf.org (W.M.); sengups@ccf.org (S.S.); 2 Department of Mathematics and Statistics, Mississippi State University, Starkville, MS 39762, USA; jshi@math.msstate.edu; 3 School of Social Work, University of North Carolina Charlotte, Charlotte, NC 28223, USA; yguo16@charlotte.edu; 4 School of Data Science, University of North Carolina Charlotte, Charlotte, NC 28223, USA; 5 School of Medicine, University of Missouri, Columbia, MO 65212, USA; ieohcz@health.missouri.edu; 6 Cleveland Clinic Lerner College of Medicine, Case Western Reserve University School of Medicine, Cleveland, OH 44195, USA; 7 Department of Quantitative Health Sciences, Cleveland Clinic, Cleveland, OH 44195, USA

**Keywords:** hepatitis C, smoking, prevalence, trends, epidemology

## Abstract

**Background**: Smoking in patients with hepatitis C (HCV) amplifies the risk of cirrhosis and hepatocellular carcinoma. We aimed to estimate the prevalence of active smoking over the last decade at the population level among adults living with HCV in the U.S., estimate temporal trends in active smoking, and identify factors associated with active smoking. **Methods**: We analyzed repeated cross-sectional NHANES data (2007–2018) of adults ≥20 years old with serologic evidence of HCV and complete smoking data (unweighted [n = 621] and weighted [n = 3,620,603] sample size). Temporal trends were evaluated using linear regression and joinpoint regression. Survey-weighted multivariable logistic regression was used to identify factors associated with active smoking. **Results**: The cumulative prevalence of active smoking was 56.4% (95% CI, 49.2–63.4). Linear trend testing was not significant (*p* = 0.93). Joinpoint regression suggested a slope change near 2013–2014, but neither segment-specific annual percent changes nor the slope change reached significance. Factors associated with higher odds of active smoking included female sex (aOR = 2.23; 95% CI, 1.17–4.24), low poverty income ratio (aOR = 3.33; 1.41–7.84), lifetime substance use (aOR = 10.63; 3.08–36.70), and depression (aOR = 2.65; 1.29–5.45). Lower odds were observed with >high-school education (aOR = 0.50; 0.26–0.94), obesity (aOR = 0.32; 0.18–0.58), and ≥2 yearly healthcare visits (aOR = 0.27; 0.10–0.68). **Conclusions**: Smoking appears to be endemic within the HCV population, and rates have remained alarmingly high and stagnant (i.e., unchanged or have not decreased) over the last decade, which consequently can lead to heightened incident cases of HCV-related cirrhosis and hepatocellular carcinoma in the near future.

## 1. Introduction

Worldwide, it is estimated that 50 million people are living with chronic hepatitis C virus (HCV), with about 1 million new cases each year and roughly 242,000 deaths due to HCV-associated cirrhosis or HCV-associated hepatocellular carcinoma [1]. In the United States, an estimated ~2.2 million adults are living with active HCV infection [2] HCV often leads to advanced liver disease, including cirrhosis, decompensated cirrhosis, and HCC.

Smoking, a behavior that alone increases the risk for cirrhosis and hepatocellular carcinoma, ref. [3] is disproportionately higher among people living with HCV compared to the general population. Smoking worsens HCV-related liver injury by increasing oxidative stress, inducing inflammatory cytokines, and activating hepatic stellate cells, which accelerates fibrosis and raises the risk for cirrhosis and hepatocellular carcinoma [4,5]. Although direct-acting antivirals have transformed HCV management, clinical guidelines recommend addressing modifiable behaviors (e.g., alcohol and tobacco) within HCV care [6].

Several studies have delineated smoking patterns among individuals infected with HCV. Utilizing the National Health and Nutrition Examination Survey (NHANES) data spanning 1999 to 2014, a study identified a substantial smoking prevalence among HCV-infected adults and elucidated socioeconomic and clinical factors associated with this behavior [7]. Complementary qualitative and mixed-methods research explored HCV-specific smoking motivations and obstacles, including stress management beliefs and inadequate utilization of cessation support mechanisms [8,9]. Another study conducted among HCV patients who inject drugs who were in opioid agonist treatment revealed exceptionally high smoking rates, which were linked to specific substance use contexts and treatment engagement patterns [10,11]. These findings collectively suggest the pervasive nature of tobacco use within HCV-affected populations, implicating socioeconomic disadvantage, mental health comorbidity, and substance use as pivotal drivers of this phenomenon. For comparative context, the prevalence of cigarette smoking among U.S. adults in 2018 was approximately 14%, while earlier NHANES analyses indicated a prevalence of around 62% among adults with HCV [12].

However, data on long-term smoking trends at the population level among people living with HCV remain limited. Many studies only provide cross-sectional snapshots of smoking prevalence or use small samples from clinical settings, and it is unknown if smoking rates have declined among adults living with HCV in the U.S. To help fill this gap at the population-level, we aimed to estimate the prevalence of active smoking over the last decade among adults living with HCV in the U.S., estimate temporal trends in active smoking, and identify demographic, clinical, and behavioral factors that predict active smoking.

## 2. Materials and Methods

### 2.1. Study Design

By combing annual data from the National Health and Nutrition Examination Survey (NHANES) cycles from 2007 to 2018, we performed a repeated cross-sectional, retrospective analysis of data. NHANES is a publicly available, de-identified database that uses a multistage, stratified probability sampling strategy to recruit a nationally representative sample of non-institutionalized adults living in the U.S. We pooled (“stacked”) six two-year waves and applied survey design variables (weights, strata, primary sampling units) per NCHS guidance to generate nationally representative estimates for adults living with HCV.

#### Sample Selection

Inclusion criteria were age ≥ 20 years, serologic evidence of HCV (anti-HCV antibody and/or HCV RNA positive per NHANES laboratory files), and non-missing smoking status. Across six waves of data (i.e., 2007–2018), 622 unweighted participants met HCV serologic criteria; one lacked smoking status and was excluded, yielding a final unweighted analytic sample of n = 621 (weighted sample size n = 3,620,603). The mean unweighted number of HCV-seropositive participants per two-year wave was 103.5 (range, 93–116). Exclusion criteria was missing data on smoking status (n = 1).

### 2.2. Primary Outcomes

The primary outcomes were the cumulative prevalence of active smoking over the last decade, trend changes in the prevalence of active smoking over the last decade, and factors that predict active smoking. Smoking status was ascertained using standardized NHANES questionnaires that assessed whether participants had smoked ≥100 cigarettes in their lifetime and whether they currently smoke cigarettes (every day or some days). Participants who answered “yes” to both questions were classified as active smokers, and all others were considered non-smokers.

### 2.3. Independent Variables

#### 2.3.1. Demographic Characteristics

Demographic variables included age, sex, ethnicity (non-Hispanic White, non-Hispanic Black, Other), relationship status (married/partnered vs. other), insurance status (insured vs. uninsured), education level (<High School [HS], High School/GED, >High School), and poverty income ratio (low vs. middle/high).

#### 2.3.2. Clinical and Laboratory Characteristics

Clinical variables encompassed obesity (body mass index ≥ 30 kg/m^2^), hypertension, hyperlipidemia, hepatitis B virus (HBV) co-infection, and advanced liver fibrosis—determined by calculating the Fibrosis-4 (FIB-4) score [age, aminotransferase (ALT), aspartate aminotransferase (AST), and platelet count were used to calculate the FIB-4 score]—serum cotinine, gamma glutamyl transferase (GGT), creatinine, and total bilirubin. HCV serology components (anti-HCV and HCV RNA) were obtained from NHANES laboratory datasets and used solely to define HCV status.

#### 2.3.3. Behavioral and Psychosocial Characteristics

Behavioral and psychosocial characteristics included current marijuana use (self-reported past 30-day use) and lifetime substance use. The Patient Health Questionnaire-9 (PHQ-9) was used to assess levels of depression, and those with a score ≥ 10 were classified as having depression.

### 2.4. Statistical Analysis

Survey design variables (weights, strata, primary sampling units) were applied to account for complex design and to obtain nationally representative estimates. Descriptive statistics included weighted means/frequencies with 95% CIs. Between-group comparisons used design-based *t*-tests (continuous) and Rao–Scott χ^2^ tests (categorical). 

To assess temporal trends in smoking prevalence (2007–2018), we first computed wave-specific weighted prevalences and then fit (i) a linear regression of prevalence on survey wave (treated as continuous) and (ii) a join-point (piecewise log-linear) regression allowing at most one join-point to avoid overfitting. Wave-level estimates were analyzed with inverse-variance weights derived from each wave’s CI on the log scale. We compared 0- vs. 1-joinpoint models using an F-test and reported annual percent change (APC) for each segment and average annual percent change (AAPC) for 2007–2018 with 95% CIs; a Davies test assessed slope change.

For determinants of active smoking, we fitted a survey-weighted binary logistic regression to the pooled sample across all waves, reporting adjusted odds ratios (aORs) with 95% CIs. Two-sided α = 0.05. Analyses used R (v4.4.1) with the survey package; join-point modeling used the segmented package. We limited trend analyses to 2007–2018 because NHANES field operations and public release structures changed during 2019–2020 (COVID-19), and the combined “2017–March 2020 pre-pandemic” files are not an independent wave for these HCV laboratory components; including them would duplicate 2017–2018 estimates and compromise trend comparability.

### 2.5. Ethical Considerations

NHANES data are publicly available and de-identified. In accordance with Cleveland Clinic IRB policy, analyses of these public-use files do not constitute human subjects research and did not require IRB review or informed consent beyond that obtained by the National Center for Health Statistics.

## 3. Results

### 3.1. Active Smoking: Prevalence and Trends

**Figure 1:**
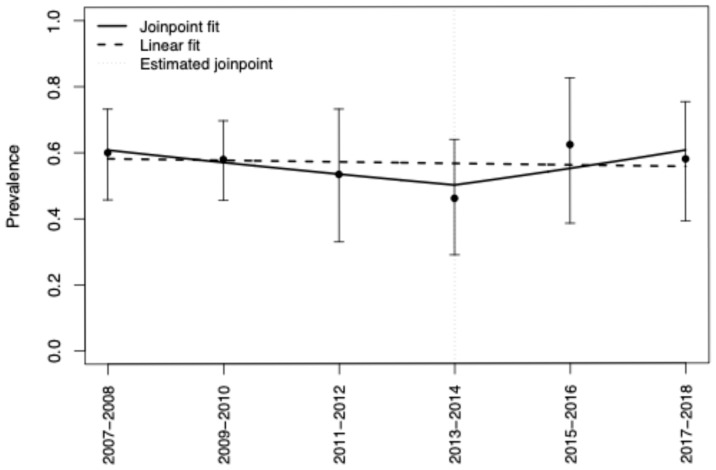
Overall Smoking Prevalence [2007–2018] and Annual Trends in the Prevalence of Active Smoking Among Adults Living with HCV in the U.S.: Joinpoint and Linear Regression Modeling. Notes: Points show survey-weighted prevalence (95% CI) for each two-year wave. The dashed line is a single-slope linear fit. The solid line is a joinpoint (piecewise log-linear) fit allowing one joinpoint; the estimated joinpoint is near 2013–2014. Neither the overall linear trend nor the slope change reached statistical significance (see Table 1 for model summaries).

Wave-specific prevalences with 95% CIs and unweighted counts are shown in (Table 1). The cumulative prevalence of active smoking among adults with HCV in the U.S. from 2007 to 2018 was 56.4% (95% CI, 49.2–63.4). Neither a simple linear model (*p* = 0.93) nor a join-point model (Davies *p* = 0.44; F-test *p* = 0.43) identified a statistically significant temporal trend; estimates suggested a decline through ~2013–2014 followed by a modest uptick, but segment-specific APCs were not different from zero (Figure 1).

### 3.2. Active Smoking: Prevalence and Trends by Demographic Characteristics

The overall cumulative prevalence of active smoking (2007–2018) was higher among adults 20–64 vs. ≥65 years old (59.4% vs. 26.9%), non-Hispanic Whites vs. non-Hispanic Blacks and those of other ethnicities (59.4% vs. 49.8% vs. 49.8%, respectively), those who were not in a relationship vs. those who were married/partnered (57.7% vs. 55.5%), those with a low vs. middle/high poverty income ratio (67.7% vs. 43.8%), those with <HS or HS/GED vs. >HS education (62.4–65.3% vs. 44.9%), and those with 0–1 vs. ≥2 yearly health care visits (73.5% vs. 49.7%) (Table 2).

Though trends in active smoking did not decrease or increase significantly across these demographic groups, certain groups had noteworthy increases in the prevalence of active smoking from 2007 to 2018. For instance, the prevalence of active smoking increased from 2007 to 2018 among those 20–64 years old (61.7% to 67.1%), non-Hispanic Blacks (41.8% to 52.9%), married/partnered (51.1% to 69.1%), with a low poverty income ratio (72.1% to 76.2%), with >HS education (41.5% vs. 49.5%), and 0–1 yearly healthcare visits (62.6% to 89.0%).

### 3.3. Active Smoking: Prevalence and Trends by Medical Conditions, Alcohol Use, Marijuana Use, and Depression

Trends in active smoking were not statistically significantly (i.e., did not increase or decrease) across any medical group, but certain groups exhibited notable upward changes from 2007 to 2018 (Table 3). The prevalence of active smoking among those with HBV co-infection increased from 52.8% to 68.5%, increased from 48.1% to 62.7% among those with advanced fibrosis, and rose from 46.0% to 72.5% among those with alcohol use and from 72.3% to 89.1% among those with current marijuana use.

### 3.4. Bivariate Analysis

Compared with non-smokers, active smokers had a higher proportion with a low poverty-income ratio (61.90% vs. 37.65%, *p* = 0.0004), a high school/GED education (37.23% vs. 25.61%, *p* = 0.0257), and depression (23.89% vs. 11.67%, *p* = 0.0084), as well as a higher mean platelet count (231 ± 7.07 vs. 208 ± 7.74, *p* = 0.0352) and serum cotinine (260.41 vs. 51.08 ng/mL, *p* < 0.001) (Table 4). Non-smokers were older on average, more likely to be married/partnered, had higher income and >high-school education, greater obesity prevalence, more diabetes, more frequent healthcare visits, and higher mean creatinine and total bilirubin.

### 3.5. Multivariate Binary Logistic Regression

In survey-weighted multivariate binary logistic regression, female sex (adjusted odds ratio [aOR] = 2.23, 95% CI 1.174.24, *p* = 0.0156), low poverty income ratio (aOR = 3.33, 95% CI 1.41–7.84, *p* = 0.0070), lifetime substance use (aOR = 10.63, 95% CI 3.08–36.70, *p* = 0.0004), and depression (aOR = 2.65, 95% CI 1.29–5.45, *p* = 0.0089) were associated with increased odds of active smoking (Table 5). Conversely, having >high school education (aOR = 0.50, 95% CI 0.26–0.94, *p* = 0.0329), obesity (aOR = 0.32, 95% CI 0.18–0.58, *p* = 0.0004), and ≥2 yearly health care visits (aOR = 0.27, 95% CI 0.10–0.68, *p* = 0.0065) were associated with lower odds of active smoking.

## 4. Discussion

In this large, population-level analysis of NHANES data over the past decade (an 11-year period) in the U.S., the prevalence of active smoking among adults living with HCV was 56.4%, and there were no significant declines in smoking over the 11-year period. Female sex, low socioeconomic status, lifetime substance use, and depression were factors associated with increased odds of active smoking, whereas greater educational attainment, obesity, and regular healthcare utilization were associated with lower odds of active smoking.

Smoking appears to be endemic within the HCV population [9,10], which further complicates management of cirrhosis and hepatocellular carcinoma. Although cigarette smoking in the general U.S. population declined from 19.8% in 2007 to 13.8% in 2018 [12], smoking rates among people living with HCV have remained alarmingly stagnant. Undoubtedly, this phenomenon will likely accelerate incident cases of HCV-related advanced liver disease, especially if high-risk subgroups (e.g., those with low income or psychiatric comorbidities) remain uninformed of the negative effects of smoking on HCV, disregard cessation advisement, or do not receive effective cessation interventions. Evidence-based pharmacotherapies—including varenicline, bupropion, and nicotine replacement therapy—have demonstrated efficacy for smoking cessation across diverse populations, including those with psychiatric comorbidities [13]. Although no large, randomized trials have specifically examined integrated HCV care in combination with smoking cessation, these medications, along with behavioral support, may hold promise for people living with HCV. Digital or text-based interventions have also shown cost-effective, scalable benefits for hard-to-reach groups and could be adapted for HCV patients. Future studies are warranted to fully assess integrated cessation interventions within HCV treatment programs.

The link between depression and active smoking may reflect a bidirectional cycle in which nicotine transiently alleviates mood disturbances (e.g., stress or anxiety) but ultimately exacerbates depressive symptoms via neurochemical or inflammatory pathways [7]. Integrating depression screening and treatment within smoking-cessation efforts could help break this cycle. Likewise, lifetime substance use often co-occurs with smoking due to shared neurobiological circuits and environmental reinforcement [10], underscoring the importance of integrated interventions that address both HCV management and substance use disorders. Another notable finding was the heightened odds of smoking among women with HCV, whereas other studies indicate men are more likely to smoke than women with HCV [14]. This divergence could be due to evolving demographic patterns, shifts in substance use trends, or previously under-recognized psychosocial factors influencing women—particularly as many older studies were cross-sectional and had smaller samples.

Smoking prevalence among adults with HCV is likely much higher than the general U.S. population due to differences in socioeconomic disadvantages, co-occurring substance and alcohol use, and psychiatric disorders. All of these respective factors are over-represented among adults with HCV and are strongly linked with tobacco use. Similarly, this is consistent with our multivariable findings.

We did notice an uptick in the 2015–2016 active smoking prevalence estimate. We re-checked wave-level sample sizes, missingness, and survey question wording and found no methodological artifacts. This interval coincided with a marked escalation of the U.S. opioid epidemic [15], tightly intertwined with HCV epidemiology via injection drug use. Smoking prevalence is exceptionally high among individuals with opioid use disorder, often exceeding 80% [16] and remains elevated among individuals with opioid use disorder (~73%), which could reinforce tobacco use within this comorbid population [17]. The observed pattern may therefore reflect broader syndemic dynamics rather than a sustained secular change. Nevertheless, our joinpoint regression modeling did not indicate a statistically significant slope change.

Given the high prevalence of undiagnosed cases of HCV in the U.S., several medical institutions have implemented universal HCV testing for all patients who present in their respective emergency departments [18]. This point of care, universal HCV testing model in the emergency department uses the SBIRT (Screening, Brief Intervention, and Referral to Treatment), which identifies undiagnosed cases of HCV, provides brief education on HCV and harmful effects of alcohol use, and refers and links patients to HCV direct-acting antiviral treatment with primary care, infectious disease, or hepatology providers. Integration of the harmful effects of smoking during the brief intervention of SBIRT and provision of tobacco cessation counseling or resources may be an optimal time to engender thoughts of behavioral change among patients at the time of diagnosis. Future studies are needed to evaluate the feasibility and impact of patient psychoeducation on the harmful, toxic hepatic effects of smoking on HCV at the time of testing and diagnosis in emergency department settings.

This study has several limitations and strengths. First, self-reported smoking—without biochemical verification may introduce recall or social desirability bias. Second, data on the number of cigarettes smoked per day were not included, limiting our ability to model changes in smoking intensity over the 11-year period. Furthermore, the absence of data on alternative tobacco products (e.g., e-cigarettes, cigars, smokeless tobacco) for this HCV-defined analytic subset precludes product-specific analyses. However, the repeated cross-sectional design across multiple NHANES cycles is significantly more robust than single cross-sectional designs. The large, nationally representative sample, use of survey weighting, and incorporation of joinpoint regression modeling enhanced both the internal and external validity of our findings.

Active smoking is still shockingly prevalent among people living with HCV, suggesting a great need for focused cessation programs addressing sex-specific risk factors, socioeconomic-driven inequalities, co-occurring substance use, and mental health issues. Future prospective studies should evaluate integrated treatment approaches that concurrently treat HCV, help smokers quit, and address psychiatric or substance use comorbidities. Such initiatives could significantly reduce morbidity and mortality, thereby improving population health and clinical care.

## 5. Conclusions

Among U.S. adults living with HCV, active smoking remains common and shows no statistically significant decline over the last decade. Integrating targeted tobacco cessation—with attention to socioeconomic disadvantage, co-occurring substance use, and depression—into HCV care pathways is an immediate actionable strategy to mitigate progression to advanced liver disease.

## Figures and Tables

**Table 1:** Prevalence of Active Smoking Among Adults ≥20 Years Old Living with HCV by NHANES Wave and Regression Summary for Trend Analysis.

**Per-Wave Prevalence (95% CI)**	
Wave	Prevalence (95% CI)
Overall 2007–2018 (n = 621)	56.4 (49.2–63.4)
2007–2008 (n = 116)	60.0 (45.7–73.2)
2009–2010 (n = 107)	57.9 (45.5–69.6)
2011–2012 (n = 93)	53.3 (32.9–73.1)
2013–2014 (n = 102)	46.0 (28.9–63.8)
2015–2016 (n = 98)	62.2 (38.4–82.4)
2017–2018 (n = 105)	57.8 (39.0–75.1)
**Linear and Joinpoint Regression Summary**	
Metric	Estimate
**Linear Regression**	
Linear trend *p*	0.9254
**Joinpoint Regression**	
Joinpoint location	~2013–2014 (95% CI ≈ 2003.5–2023.5)
Trend 1 (years)	2007–2014
Trend 1 APC (95% CI)	−3.20%/yr (95% CI −9.11, 3.11)
Trend 2 (years)	2014–2018
Trend 2 APC (95% CI)	+4.86%/yr (95% CI −2.66, 12.96)
AAPC 2007–2018	−0.05%/yr

Notes: APC = annual percent change; AAPC = average annual percent change; CI = Confidence Interval. Joinpoint model fit on log (prevalence) with ≤1 joinpoint (to avoid overfitting); slope-change tests were not significant (Davies *p* = 0.4422; F-test *p* = 0.4311).

**Table 2:** Trends in the Prevalence of Active Smoking Among Adults ≥20 Years Old Living with HCV—Stratified by Demographics: NHANES 2007–2008 to 2017–2018.

Variables	2007–2018	NHANES 2007–2008	NHANES 2009–2010	NHANES 2011–2012	NHANES 2013–2014	NHANES 2015–2016	NHANES 2017–2018	Trend *p*
**Sex**								
**Female**	58.9 (48.2–69.0)	58.8 (37.0–78.4)	56.8 (33.5–78.2)	44.3 (11.6–81.2)	64.2 (37.8–85.7)	77.3 (50.5–94.0)	55.3 (27.1–81.2)	0.5458
**Male**	55.0 (46.4–63.4)	60.8 (43.6–76.3)	58.3 (45.8–70.0)	59.1 (35.8–79.9)	35.4 (14.2–61.6)	54.4 (28.8–78.5)	59.5 (36.5–79.9)	0.6579
**Age Group**								
**20–64**	59.4 (51.3–67.2)	61.7 (47.3–74.8)	59.4 (46.9–71.1)	53.4 (31.6–74.3)	48.4 (28.6–68.6)	63.5 (39.8–83.3)	67.1 (40.0–87.9)	0.6100
**≥65**	26.9 (14.2–42.9)	16.7 (0.0–89.9)	14.7 (0.0–75.1)	52.8 (4.4–97.0)	30.5 (5.9–67.7)	48.7 (6.5–92.5)	12.2 (0.2–53.9)	0.7460
**Ethnicity**								
**Non-Hispanic White**	59.4 (49.6–68.7)	64.8 (47.5–79.9)	60.3 (39.6–78.7)	51.7 (23.8–78.8)	49.7 (22.2–77.4)	69.3 (35.3–92.7)	59.2 (26.0–87.2)	0.9679
**Non-Hispanic Black**	49.8 (41.9–57.7)	41.8 (15.5–72.0)	58.6 (36.6–78.4)	53.7 (29.4–76.8)	38.9 (20.0–60.5)	56.6 (34.7–76.8)	52.9 (25.9–78.7)	0.6630
**Other**	49.8 (39.8–59.9)	65.6 (38.4–87.1)	47.9 (28.9–67.3)	62.9 (17.6–95.3)	41.8 (26.4–58.4)	35.7 (14.6–61.5)	54.2 (18.2–87.3)	0.2893
**Relationship status**								
**Married/partnered**	55.5 (44.4–66.3)	51.1 (29.4–72.6)	54.0 (35.6–71.7)	47.9 (19.1–77.8)	49.1 (25.4–73.1)	53.4 (23.3–81.7)	69.1 (34.7–92.7)	0.1900
**Other**	57.3 (47.6–66.6)	70.8 (53.2–85.0)	61.9 (47.7–74.9)	61.4 (40.6–79.6)	44.0 (17.4–73.3)	67.1 (35.9–90.2)	41.7 (21.6–64.0)	0.1628
**Health insurance**	48.7 (40.7–56.8)	52.7 (41.3–63.9)	41.1 (25.1–58.6)	47.5 (27.9–67.6)	41.8 (25.8–59.1)	53.9 (30.1–76.5)	51.3 (30.3–71.9)	0.6364
**Public insurance**	55.8 (46.0–65.3)	63.5 (47.0–78.0)	62.5 (37.1–83.8)	56.2 (30.8–79.6)	52.8 (32.3–72.7)	60.4 (44.8–74.7)	50.2 (22.9–77.3)	0.0842
**Poverty income ratio**								
**low**	67.7 (59.4–75.3)	72.1 (61.0–81.7)	74.0 (56.5–87.4)	53.4 (24.6–80.7)	58.0 (34.9–78.8)	68.0 (50.3–82.8)	76.2 (53.4–91.7)	0.9384
**middle or high**	43.8 (33.4–54.5)	43.1 (21.3–66.9)	41.0 (19.2–65.6)	52.8 (21.3–82.7)	31.7 (11.4–58.6)	55.6 (21.5–86.2)	37.8 (13.5–67.4)	0.9664
**Education**								
**<HS**	62.4 (52.6–71.5)	67.5 (46.9–84.2)	72.2 (53.5–86.8)	74.6 (38.0–95.9)	33.7 (10.2–64.9)	64.6 (41.3–83.9)	51.8 (0.0–100.0)	0.3220
**HS graduate/GED**	65.3 (55.5–74.2)	69.1 (49.5–84.8)	68.9 (52.3–82.8)	83.7 (46.3–98.9)	53.3 (32.0–73.8)	63.1 (6.0–99.2)	64.8 (38.4–86.1)	0.4666
**>HS graduate**	44.9 (34.3–55.7)	41.5 (18.8–67.1)	42.6 (22.1–65.1)	30.0 (13.8–50.7)	46.5 (20.0–74.4)	59.9 (25.5–88.4)	49.5 (18.4–80.9)	0.2208
**Number of health care visits**								
**0–1 yearly visits**	73.5 (60.0–84.6)	62.6 (26.7–90.5)	74.3 (43.3–94.0)	68.1 (11.3–99.1)	48.9 (11.7–87.2)	85.7 (48.7–99.3)	89.0 (60.3–99.3)	0.2841
**≥2 yearly visits**	49.7 (41.7–57.8)	59.1 (43.3–73.8)	47.3 (31.1–63.8)	44.1 (25.0–64.4)	45.4 (28.2–63.4)	52.5 (27.7–76.4)	49.2 (26.7–72.0)	0.5417

Abbreviations: NHANES = National Healthy and Nutrition Examination Survey; HS = High School; GED = General Educational Development (i.e., high school diploma). Notes: Data displayed as weighted percentages (95% confidence interval) except where otherwise noted; Test for trends was performed by including the prevalence of each survey period as a continuous variable in a linear regression model. To avoid issues with calculating confidence intervals based on small sample sizes, we binned some subgroups (e.g., age, ethnicity, poverty income ratio, and number of health care visits).

**Table 3:** Trends in the Prevalence of Active Smoking Among Adults ≥20 Years Old Living with HCV—Stratified by Medical Conditions, and Alcohol Use, Marijuana Use, and Depression: NHANES 2007–2008 to 2017–2018.

Variables	2007–2018	NHANES 2007–2008	NHANES 2009–2010	NHANES 2011–2012	NHANES 2013–2014	NHANES 2015–2016	NHANES 2017–2018	Trend *p*
**Hepatitis B (HBV)**	56.4 (43.7–68.5)	52.8 (23.7–80.5)	58.1 (23.6–87.5)	38.3 (8.2–77.1)	46.3 (12.4–83.0)	69.3 (33.0–93.6)	68.5 (24.1–96.3)	0.2873
**Obesity**	36.6 (25.4–48.8)	49.1 (17.9–80.9)	48.5 (18.4–79.5)	29.3 (17.2–43.7)	34.6 (9.3–68.4)	39.2 (8.1–78.7)	26.6 (3.8–65.9)	0.0794
**Hypertension**	56.7 (47.9–65.3)	57.0 (36.4–76.1)	53.9 (36.6–70.7)	50.9 (29.0–72.5)	53.4 (28.8–77.0)	69.2 (49.3–85.2)	56.1 (33.6–77.0)	0.4796
**Hyperlipidemia**	53.2 (39.8–66.4)	70.1 (54.5–83.0)	45.7 (17.1–76.5)	54.1 (13.0–91.3)	48.2 (13.2–84.6)	58.8 (16.9–92.7)	46.7 (12.9–83.0)	0.3383
**Advanced fibrosis**	56.8 (41.7–71.1)	48.1 (22.0–74.9)	66.6 (41.6–86.4)	46.0 (17.3–76.8)	52.8 (11.1–91.6)	68.9 (19.0–97.9)	62.7 (7.4–98.8)	0.3451
**Alcohol Use**								
**Non-drinker**	50.7 (38.7–62.8)	46.0 (25.9–67.0)	58.1 (30.2–82.7)	70.0 (27.0–96.2)	21.0 (3.3–54.2)	30.6 (10.0–58.9)	72.5 (19.8–98.8)	0.9962
**Drinker**	58.7 (49.8–67.2)	74.2 (55.0–88.6)	55.4 (39.0–71.0)	46.9 (20.1–75.1)	58.6 (36.2–78.8)	74.8 (51.3–91.2)	51.9 (29.4–74.0)	0.7176
**Current marijuana use**	71.2 (54.8–84.6)	72.3 (41.5–92.8)	77.4 (30.6–98.7)	55.8 (6.6–96.8)	59.5 (2.0–99.7)	64.8 (5.5–99.5)	89.1 (62.9–99.1)	0.6812
**Depressed**	72.5 (61.1–82.3)	74.8 (45.9–93.4)	67.2 (36.6–89.9)	81.6 (31.8–99.6)	82.3 (43.2–98.8)	80.0 (47.0–97.1)	45.3 (3.2–94.2)	0.4216

Abbreviations: NHANES = National Healthy and Nutrition Examination Survey. Notes: Data displayed as weighted percentages (95% confidence interval) except where otherwise noted. Test for trends was performed by including the prevalence of each survey period as a continuous variable in a linear regression model. Diabetes and cancer history variables were not included due to small sample sizes within subgroups for which a weighted confidence interval could not be calculated.

**Table 4:** Demographic and Clinical Characteristics of Adults Living with HCV With and Without Active Smoking: NHANES 2007–2008 to 2017–2018.

Characteristic	Adults with HCV Who with Active Smoking	Adults with HCV Without Active Smoking	*p*-Value	Total Adults
	(n = 2,042,081)	(n = 1,578,522)		(n = 3,620,603)
**Demographics**				
**Age, mean (SE)**	49.71 (0.81)	55.37 (0.74)	0.0000	52.18 (0.59)
**Age, %**				
**20–29**	4.00 (0.01)	1.61 (0.01)	0.1386	2.96 (0.01)
**30–44**	22.90 (0.03)	9.36 (0.02)	0.0021	17.00 (0.02)
**45–64**	68.67 (0.04)	73.49 (0.04)	0.4549	70.77 (0.02)
**≥65**	4.43 (0.01)	15.54 (0.04)	0.0004	9.27 (0.02)
**Sex, %**			0.5267	
**Female**	38.12 (0.03)	34.45 (0.05)		36.52 (0.03)
**Male**	61.88 (0.03)	65.55 (0.05)		63.48 (0.03)
**Ethnicity, %**				
**Non-Hispanic White**	72.56 (0.03)	64.23 (0.04)	0.0702	68.93 (0.03)
**Non-Hispanic Black**	13.69 (0.02)	17.88 (0.02)	0.1570	15.52 (0.02)
**Mexican American**	4.59 (0.01)	6.61 (0.02)	0.2321	5.47 (0.01)
**Other Hispanic**	4.69 (0.01)	5.76 (0.01)	0.4810	5.16 (0.01)
**Other Race**	4.46 (0.02)	5.54 (0.02)	0.6289	4.93 (0.01)
**Relationship status, %**			0.8147	
**Married/partnered**	49.82 (0.04)	51.59 (0.05)		50.59 (0.03)
**Health insurance, %**			0.0004	
**Yes**	60.47 (0.04)	82.28 (0.04)		69.98 (0.03)
**Poverty income ratio (income level), %**				
**index ≤ 1.30 (low)**	61.90 (0.04)	37.65 (0.04)	0.0004	51.24 (0.03)
**1.30 < index ≤ 1.85 (middle)**	10.89 (0.02)	11.62 (0.03)	0.8378	11.21 (0.02)
**index > 1.85 (high)**	27.21 (0.04)	50.73 (0.05)	0.0016	37.55 (0.03)
**Education, %**				
**<HS**	31.35 (0.04)	24.47 (0.03)	0.1359	28.35 (0.03)
**HS graduate/GED**	37.23 (0.04)	25.61 (0.04)	0.0257	32.16 (0.03)
**>HS graduate**	31.42 (0.04)	49.93 (0.05)	0.0014	39.49 (0.03)
**Alcohol Use, Substance Use, and Psychiatric Characteristics**				
**Active Drinker, %**			0.2613	
**Non-drinker**	29.59 (0.05)	36.69 (0.04)		32.71 (0.04)
**Drinker** **(currently drinking heavily and not heavily)**	70.41 (0.05)	63.31 (0.04)		67.30 (0.04)
**Current marijuana use, %**	39.34 (0.05)	26.85 (0.07)	0.1712	34.70 (0.04)
**Current cocaine use, %**	18.63 (0.05)	8.36 (0.06)	0.2401	15.19 (0.04)
**Lifetime substance use**	96.51 (0.01)	87.77 (0.03)	0.0053	93.02 (0.01)
**PHQ-9 Depression Score, mean (SE)**	5.99 (0.48)	4.20 (0.38)	0.0080	5.21 (0.29)
**Depressed, %**	23.89 (0.03)	11.67 (0.02)	0.0084	18.55 (0.02)
**Clinical Characteristics**				
**BMI, mean (SE)**	25.77 (0.39)	29.19 (0.60)	0.0000	27.26 (0.41)
**Obesity, %**	16.40 (0.03)	37.05 (0.04)	0.0001	25.37 (0.03)
**Hypertension, %**	43.30 (0.05)	46.23 (0.04)	0.6345	44.52 (0.03)
**Hyperlipidemia, %**	30.63 (0.04)	34.56 (0.05)	0.5311	32.35 (0.03)
**Diabetes (T2DM), %**	11.06 (0.03)	24.35 (0.06)	0.0295	16.06 (0.03)
**Hepatitis B (HBV), %**	31.89 (0.04)	31.20 (0.04)	0.8981	31.59 (0.03)
**History of Cancer, %**	7.65 (0.02)	14.31 (0.04)	0.0500	10.56 (0.02)
**Number of Health Care Visits, %**				
**None**	20.21 (0.04)	10.80 (0.03)	0.0828	16.11 (0.02)
**1**	16.33 (0.02)	6.23 (0.02)	0.0016	11.93 (0.02)
**2 to 3**	18.38 (0.03)	27.11 (0.04)	0.0506	22.19 (0.03)
**4 to 9**	24.03 (0.04)	34.82 (0.05)	0.0921	28.74 (0.03)
**10 to 12**	10.62 (0.03)	8.23 (0.02)	0.5125	9.58 (0.02)
**≥13**	10.41 (0.02)	12.80 (0.03)	0.4900	11.45 (0.02)
**Number of Health Care Visits, %**			0.0034	
**0–1 yearly visits**	36.55 (0.04)	17.03 (0.04)		28.04 (0.03)
**≥2 yearly visits**	63.45 (0.04)	82.97 (0.04)		71.96 (0.03)
**Liver-Related Clinical Characteristics**				
**Alanine Transaminase (ALT) (U/L), mean (SE)**	54.24 (4.94)	44.68 (2.64)	0.0621	50.05 (3.27)
**Aspartate Transaminase (AST) (U/L), mean (SE)**	51.15 (3.42)	45.41 (3.48)	0.1766	48.63 (2.73)
**Platelet Count (1000 cells/uL), mean (SE)**	230.88 (7.07)	208.17 (7.74)	0.0352	220.97 (5.48)
**Serum Cotinine Levels (ng/mL), mean (SE)**	260.41 (12.46)	51.08 (13.57)	0.0000	168.58 (10.96)
**Gamma Glutamyl Transferase (GGT) (U/L),** **mean (SE)**	63.83 (4.86)	67.78 (6.89)	0.6377	65.56 (4.13)
**Creatinine (mg/dL), mean (SE)**	0.85 (0.01)	0.93 (0.02)	0.0008	0.89 (0.01)
**Total Bilirubin (mg/dL)**	0.62 (0.03)	0.72 (0.05)	0.0489	0.66 (0.03)
**FIB-4 Score, mean (SE)**	2.02 (0.20)	2.40 (0.32)	0.2789	2.19 (0.20)
**Advanced Fibrosis (FIB-4), %**	19.92 (0.04)	19.52 (0.04)	0.9359	19.74 (0.03)

Abbreviations: NHANES = National Health and Nutrition Examination Survey SE = standard error; HS = high school; BMI = body mass index; FIB-4 = Fibrosis-4 Index. Note: Data displayed as weighted mean/percent (SE) except where otherwise noted.

**Table 5:** Weighted Multivariate Binary Logistic Regression: Active Smoking.

Variable	aOR	95% CI		*p*-Value
**(Intercept)**	1.04	0.13	8.56	0.9670
**Age**	0.97	0.94	1.01	0.1219
**Sex, %**				
**Female (Ref.: Male)**	2.23	1.17	4.24	0.0156
**Ethnicity, %**				
**Non-Hispanic Black (Ref: Non-Hispanic White)**	0.86	0.44	1.71	0.6684
**Mexican American (Ref: Non-Hispanic White)**	0.66	0.25	1.71	0.3834
**Other Hispanic (Ref: Non-Hispanic White)**	0.60	0.17	2.10	0.4177
**Other Race (Ref: Non-Hispanic White)**	0.74	0.18	3.00	0.6652
**Health insurance (Ref: No)**	0.73	0.36	1.49	0.3804
**Poverty income ratio (income level)**				
**index ≤ 1.30 [low] (Ref: index > 1.85 [high])**	3.33	1.41	7.84	0.0070
**1.30 < index ≤ 1.85 [middle] (Ref: index > 1.85 [high])**	1.79	0.65	4.88	0.2500
**Education**				
**HS graduate/GED (Ref: (<HS)**	1.59	0.79	3.18	0.1849
**>HS graduate (Ref: <HS)**	0.50	0.26	0.94	0.0329
**Active Drinker (Ref: No)**	0.97	0.51	1.84	0.9246
**Lifetime substance use (Ref: No)**	10.63	3.08	36.70	0.0004
**Depressed (Ref: No)**	2.65	1.29	5.45	0.0089
**Obesity (Ref: No)**	0.32	0.18	0.58	0.0004
**History of Cancer (Ref: No)**	0.66	0.20	2.23	0.4941
**Number of Health Care Visits**				
**≥2 yearly visits (Ref: 0–1 yearly visits)**	0.27	0.10	0.68	0.0065
**Wave**				
**2009–2010 (Ref: 2007–2008)**	0.98	0.46	2.08	0.9645
**2011–2012 (Ref: 2007–2008)**	0.91	0.26	3.20	0.8823
**2013–2014 (Ref: 2007–2008)**	0.85	0.29	2.50	0.7565
**2015–2016 (Ref: 2007–2008)**	1.24	0.48	3.17	0.6514
**2017–2018 (Ref: 2007–2008)**	1.30	0.56	3.04	0.5301

Abbreviations: aOR = adjusted odds ratio; CI = confidence interval; Ref = reference; HS = High School; GED = General Educational Development (i.e., high school diploma).

## Data Availability

All data underpinning this study are openly available from the National Health and Nutrition Examination Survey (NHANES) repository at https://www.cdc.gov/nchs/nhanes/ (accessed on 17 October 2024).

## Abbreviations

The following abbreviations are used in this manuscript:

NHANES	National Health and Nutrition Examination Survey
HCV	Hepatitis C Virus
CI	Confidence Interval
OR/aOR	(Adjusted) Odds Ratio
NIAAA	National Institute on Alcohol Abuse and Alcoholism
APC	Article Processing Charge
